# Syntheses and structures of dinuclear zinc(II) acetate-bridged coordination compounds with the aromatic Schiff base chelators *N*,*N*-dimethyl-2-[phen­yl(pyridin-2-yl)methyl­idene]hydrazine-1-carbothio­amide and *N*-ethyl-2-[phen­yl(pyridin-2-yl)methyl­idene]hydrazine-1-carbo­thio­amide

**DOI:** 10.1107/S2056989025005407

**Published:** 2025-06-24

**Authors:** Christian S. Parry, Alex R. Abraham, Samuel K. Kwofie, Michael D. Wilson, Timothy R. Ramadhar, Raymond J. Butcher

**Affiliations:** a Department of Microbiology College of Medicine Howard University, Washington, DC 20059 USA; b Department of Chemistry College of Arts and Science Howard University, Washington, DC 20059 USA; c Department of Biomedical Engineering, School of Engineering Sciences, College of Basic and Applied Sciences, University of Ghana, Legon, Accra, LG 77, Ghana; d Department of Parasitology, Noguchi Memorial Institute for Medical Research, College of Health Sciences, University of Ghana, Legon, Accra, LG 581, Ghana; University of Aberdeen, United Kingdom

**Keywords:** crystal structure, mobile zinc, lipophilicity, mixed donor set, coordination chemistry, cell biology

## Abstract

In the centrosymmetric title complexes, di-μ-acetato-bis({*N*,*N*-dimethyl-2-[phenyl(pyridin-2-yl)methylidene]hydrazine-1-carbothioamidato}zinc(II)), [Zn_2_(C_15_H_15_N_4_S)_2_(C_2_H_3_O_2_)_2_] (**I**), and di-μ-acetato-bis({*N*-ethyl-2-[phenyl(pyridin-2-yl)methylidene]hydrazine-1-carbothioamidato}zinc(II)), [Zn_2_(C_16_H_17_N_4_S)_2_(C_2_H_3_O_2_)_2_] (**II**), the zinc ions are chelated by the *N*,*N*,*S*-tridentate ligands and bridged by pairs of acetate ions. The acetate ion in (**I**) is disordered over two orientations in a 0.756 (6):0.244 (6) ratio, leading to different zinc coordination modes for the major (5-coordinate) and minor (6-coordinate) disorder components. Geometrical indices [τ_5_ = 0.32 and 0.30 for (**I**) (major component) and (**II**), respectively] suggest the zinc coordination in these phases to be distorted square pyramidal. This study forms part of our aim to discern the mechanism of metal binding in these chelators, their specificity and selectivity, and to gain insight into the role of cellular zinc in physiological processes such as infection, immunity and cancer.

## Abstract 2

Both ligands bind to zinc in an *N*,*N*,*S*-tridentate manner and form centrosymmetric dimers *via* bridging by acetate moieties. Unexpectedly, we found three distinct modes of metal coordination from the acetate O atoms. These results are discussed in the context of the vital role of zinc in cell metabolism.

## 1. Chemical context

Divalent zinc (Zn^2+^) is a highly abundant and essential nutrient in the human body and is required in nearly all cellular function including cell growth, DNA repair, and immune function (Berg & Shi, 1996 ▸; Lonergan & Skaar, 2019 ▸). Zinc is important in pharmacology, toxicology and in imaging as cellular probes (Pluth *et al.*, 2011 ▸; Radford & Lippard, 2013 ▸). Like iron, both an excess and a deficiency of zinc lead to cellular and organism-level pathology. It is therefore necessary that the levels and distribution of labile zinc be exquisitely regulated within and outside the cell.

Comparatively, a lot is known about the role of zinc in proteins, as exemplified by the zinc finger structural motif (Frankel *et al.*, 1987 ▸; Berg, 1990 ▸). This, however, may have overshadowed other essential roles of zinc, thus limiting our understanding of cell biology and our ability to design ligands that are able to modulate cellular homeostasis. There is a sizeable pool of ‘free’ or ‘labile zinc’ – non-protein bound zinc attached to a vast number of low mol­ecular weight ligands – that take part in ligand binding and ligand exchange within and outside the cell. There is a need to investigate the function of labile zinc within cells and tissues. This requires tools that can detect ’free’ zinc ion species in a qu­anti­tative manner, reporting their exact cellular location and precise inter­action.

Zinc chelators are such tools, but they have not been well studied (Dean *et al.*, 2012 ▸). Zinc chelators are important for zinc ion sequestration and transport, and can be used as probes for imaging. Zinc-chelating agents can be designed with respect to affinity, hydro­phobicity, lipophilicity and specificity for diverse metals – the basis of zinc preference for donor atoms and coordination chemistry. Current zinc probes lack specificity and may also have side effects in living systems (Krężel & Maret, 2016 ▸; Catapano *et al.*, 2018 ▸). The search for chelators of improved efficacy with no side effects remains a distant goal.

Chelating ligands have therapeutic and diagnostic use in the clinic and in research. The biological activities of metal-bound ligand complexes differ from those of either the ligand or the metal ion itself, and increased or decreased biological activity has been reported for several transition metal complexes. Of clinical inter­est, Richardson and coworkers have demonstrated that the Schiff base ligand series 2-(di-2-pyridinyl­methyl­ene)-*N*,*N*-dimethyl-hydrazinecarbo­thio­amide (Dp44mT), *N*,*N*-dimethyl-2-[phen­yl(pyridin-2-yl)methyl­idene]hydra­zine­carbo­thio­amide (Bp44mT), and di-2-pyridyl­ketone 4-cyclo­hexyl-4-methyl-3-thio­semicarbazone (DpC) used as iron chelators have strong anti-tumor response (Yuan *et al.*, 2004 ▸; Yu *et al.*, 2011 ▸; Heffeter *et al.*, 2019 ▸). Bp44mT (neutral mol­ecule C_15_H_16_N_4_S, anion C_15_H_1_5N_4_S^−^) is the ligand, **L1**. Richardson and colleagues also showed that these chelators in complex with metals have additional properties and are able to overcome clinical drug resistance. Specifically, the zinc complexes of Dp44mT, DpC and **L1** have potent cytotoxic activity against cancer cells and are able to target the lysosome through transmetallation with copper (Yu *et al.*, 2012 ▸; Sestak *et al.*, 2015 ▸; Stacy *et al.*, 2016 ▸). Metals, especially iron and zinc, are also crucial at the inter­section of immunology and infectious diseases (Weinberg, 1984 ▸; Cassat & Skaar, 2013 ▸; Nairz & Weiss, 2020 ▸). Their coordination chemistry and stereochemistry are important with respect to their transport and recognition in the microbial niche (Winkelmann & Braun, 1981 ▸; Adjimani & Emery, 1988 ▸; Juttukonda *et al.*, 2020 ▸). Recently, Skaar and coworkers have shown convincingly that dietary zinc deficiency critically degrades the immune response against pneumonia and promotes Acinetobacter baumannii lung infection in elders and in patients who require ventilation (Palmer *et al.*, 2024 ▸).

The many excellent biological attributes of Zn^2+^ ion derive from its electronic structure as a 3*d*^10^ ion. As such, zinc lacks ligand field stabilization energy or preference for a specific geometry. Zinc has coordination flexibility that facilitates rapid adoption of different structural geometries depending on the ligand and the environment – the electrostatic and steric inter­actions around the ligands – and not by the ion’s electronic ligand field stabilization energy. This also facilitates rapid ligand exchange. These properties endow zinc with its adaptability enabling it to participate in many biological functions and rapidly with diverse coordination and hapticity (Krężel & Maret, 2016 ▸). When zinc is penta-coordinate, it may adopt either a trigonal–bipyramidal or square-pyramidal structure. Also, the filled *d* orbitals precludes it from taking part in redox reactions. Zinc has a single normal oxidation state (+2) and the zinc ion only functions as a Lewis acid, a property crucial for its buffering and anti­oxidant role in the cell (Krężel & Maret, 2016 ▸). Biological zinc is predominantly coordinated by nitro­gen donor atoms (as in histidine), sulfur donor atoms (as in cysteine residues), and with O donor atoms, as in glutamate or aspartate (Karlin *et al.*, 1997 ▸).

We recently described a more and highly effective derivative chelating agent, the ligand (*E*)-*N*-ethyl-2-(phen­yl(pyridin-2-yl)methyl­ene)hydrazine-1-carbo­thio­amide (neutral mol­ecule C_16_H_18_N_3_S, anion C_16_H_17_N_3_S^−^) (**L2**). **L2** is commonly called 2-phenyl-1-pyridin-2-yl-ethanone or PPYeT (Kumari *et al.*, 2012 ▸) and is built on the common existing thio­semicarbazone (TSC) backbone (Parry *et al.*, 2025 ▸; Bonaccorso *et al.*, 2019 ▸), after ligand **L1** (Yu *et al.*, 2012 ▸). **L2** has a more flexible scaffold compared to previously reported thio­semicarbazone BpT-based chelators and was more specific and had greater chelating effectiveness with fewer side effects (Kumari *et al.*, 2012 ▸). **L2** has also shown unusual effectiveness as an anti­viral agent. The reported efficacy and desirable properties have spurred us to carry out detailed structural analyses of this new class of metal chelators. To that end, we have prepared the respective zinc compounds of **L1** and **L2** [(**I)** and (**II**), respectively] to gain insight to their structure, metal-bound complexes and coordination chemistry to elucidate their mechanism, the basis of their specificity and selectivity, and to expand their use.
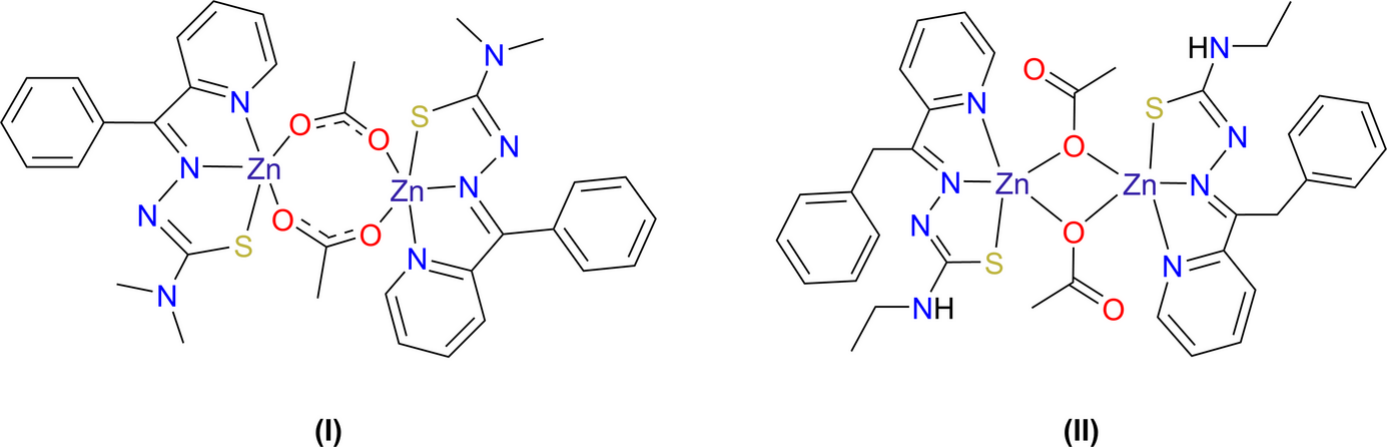


## 2. Structural commentary

The crystal structure of the reaction product of zinc-ion binding to ligand **L1** is a 2:2 complex (**I**), a dimer of monomeric zinc-bound ligands. Likewise, the structure of the reaction product of zinc and **L2** is a 2:2 complex (**II**). In both compounds, the organic ligand is bound to zinc in a tridentate fashion through the *N*,*N*′,*S* donor set, as expected. Further, the two metal ions in the complex are bridged by two acetate linkers to form 2:2 complexes. Selected geometrical data are listed in Tables 1 ▸ and 2 ▸.

**Figure 1:**
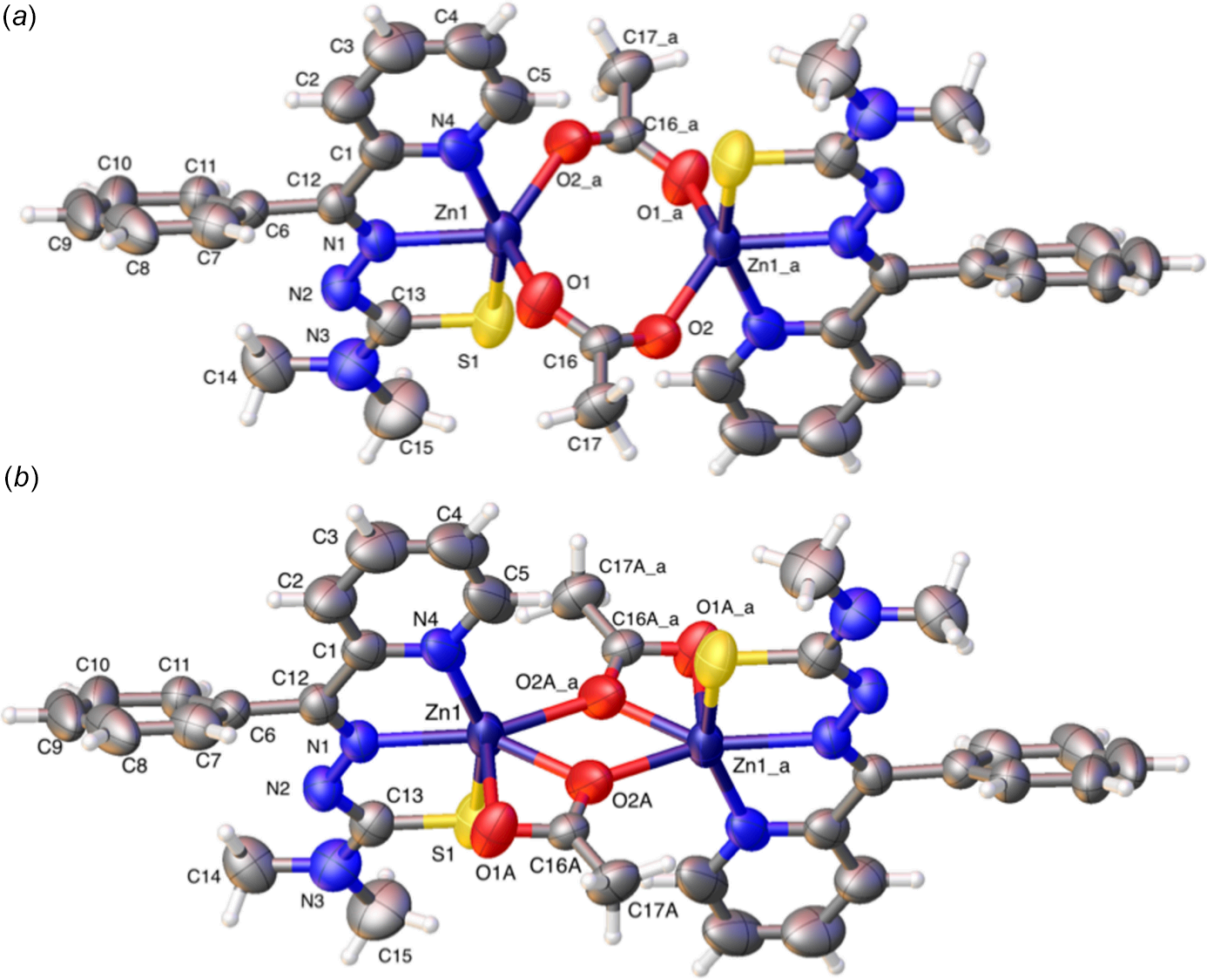
Disordered structure of (**I**): (*a*) the major disorder component, in which the zinc ion binds to ligand donors in 5-coordinate mode; (*b*) the minor disorder component, in which the zinc atom binds in a six-coordinate mode. Atoms with suffix a are generated by the symmetry operation −*x*, 1 − *y*, 1 − *z*.

**Figure 2:**
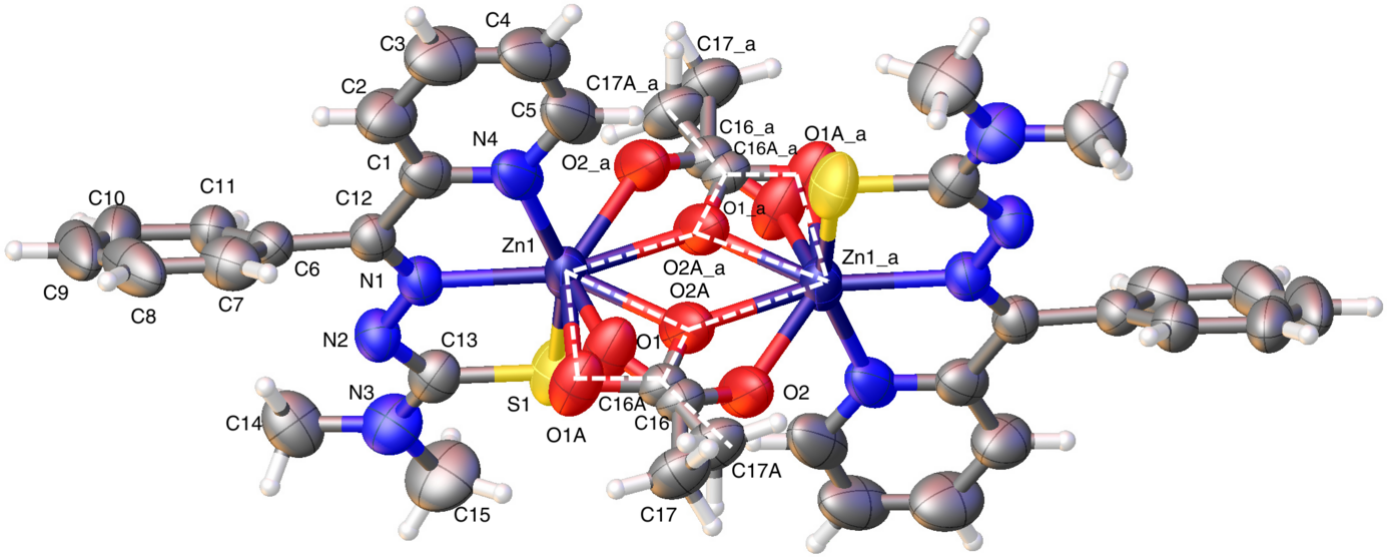
Overlay of the major and minor components of (**I**). Coordinating bonds of the minor component are shown in dashed lines in white. Atoms with suffix a are generated by the symmetry operation −*x*, 1 − *y*, 1 − *z*.

We encountered disorder of the acetate ion in (**I**) during refinement. The disorder was modeled with two equivalent orientations (Müller *et al.*, 2006 ▸; Herbst-Irmer, 2016 ▸; Archana *et al.*, 2022 ▸). The major domain was assigned 76% occupancy; this is the orientation described above as five-coordinate (Fig. 1 ▸*a*). The alternate domain has a zinc metal center coordinating, as previously described, with the ligand anion through the *N*,*N*′,*S* donor set but with additional coordinate bonds to both oxygen atoms of an acetate linker and to a single O atom from the second acetate linker, so that each zinc center altogether forms a six-coordinate geometry (24% occupancy) (Fig. 1 ▸*b*). The two domains together, superimposed as in the crystal, are shown in Fig. 2 ▸, with zinc-coordinating bonds of the minor domain shown with dashed lines in white. A tilt of the C—C stem (bond C16*A*—C17*A*) of the acetate group can also be seen.

**Figure 3:**
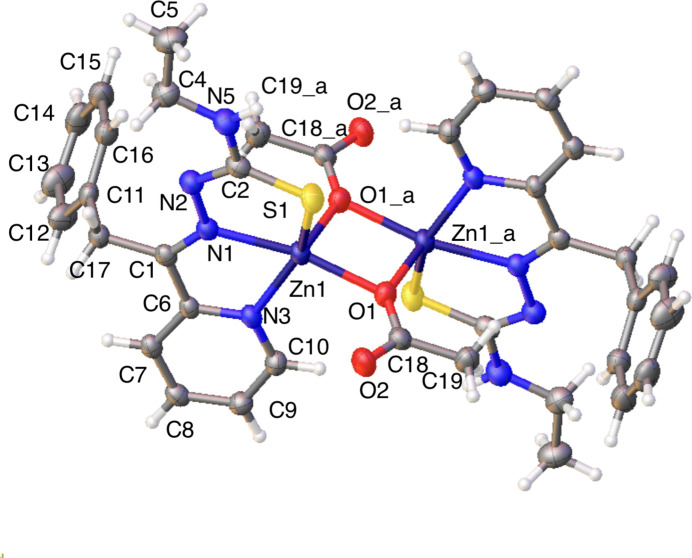
The mol­ecular structure of (**II**). Atoms with suffix a are generated by the symmetry operation −*x*, 1 − *y*, 1 − *z*.

The structure of (**II**), also a 2:2 complex, on the other hand, was not twinned. In this structure, a zinc ion coordinates the N,N′,S donor set of the **L2** anionic ligand in (**II**) and with two oxygen atoms: one O atom from each of the two acetate linkers, in penta-coordinate mode. The other zinc ion makes similar coordination with the mixed donor set. In distinct contrast with either of the two zinc coordination modes seen in complex (**I**), in complex (**II**), one O atom in the acetate linker is left uncoordinated (Fig. 3 ▸).

**Figure 4:**
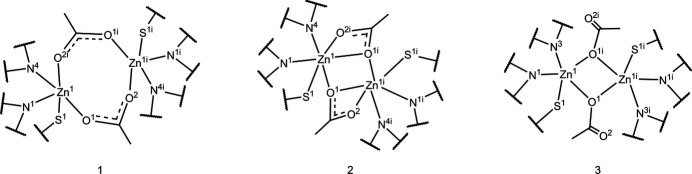
The coordination modes of the zinc centers in the metal-bound complexes. Three distinct coordination modes are discernible in our analysis.

Therefore, from the two crystal structures, we find three distinct zinc coordination modes (Fig. 4 ▸). Modes 1 and 2 correspond to the major and minor domains of complex (**I**) (Fig. 1 ▸; panels *a* and *b*, respectively), and mode 3 corresponds to the sole structure of (**II**).

A notable feature of Zn^2+^ ions is inducing dimerization. Dimer formation would be favored especially in the context of heterocyclic ligands such as **L1** and **L2** presenting with a mixed donor set and the carboxyl­ate group from the metal salt serving as a bridge. Dimerization allows the formation of more ordered structures with greater stability and enhanced functional efficacy including cooperative binding. Zinc ion-induced dimerization is common in proteins; examples are zinc fingers and class II major histocompatibility complex mol­ecules (Wang *et al.*, 2001 ▸; Li *et al.*, 2007 ▸). Zinc ion-induced dimerization is also important in small mol­ecule ligand inter­actions in cells and tissues.

Comparing the complexed structures in this study, the zinc coordinate bonds appear to be shorter in (**II**) than in (**I**): for example, the zinc–pyridine N bond length in (**II**), Zn1—N3 = 2.113 (2) Å is perceptibly shorter than in (**I**) [2.159 (5) Å]. This trend is true for zinc coordination to the donors within the ligand (pyridine N, imine N and sulfur S1) (Tables 1 ▸ and 2 ▸). Zinc coordinate bonding bridging the O atoms from the acetate anion remain tight in complex (**II**) [Zn1—O1^i^; symmetry code: (i) −*x*, −*y* + 1, −*z* + 1, Zn1—O1 average bond length = 2.055 Å]. Comparable bond lengths in complex (**I**) *major* domain are: Zn1—O2^i^ = 2.006 (5) Å and Zn1—O1 = 2.018 (5) Å indicating there is strong bonding through the bridging O atoms in the major disorder component of (**I**). The corresponding bond lengths in the minor component are Zn1—O2*A*^i^ = 2.410 (18) Å, Zn1—O1*A*^i^ = 2.052 (18) Å and Zn1—O1*A* = 2.114 (14) Å (Tables 1 ▸ and 2 ▸).

In the major component of (**I**), the zinc center makes a coordinate bond with an O atom from each acetate group; the angle at the zinc center, O2^i^—Zn1—O1, is 121.0 (3)°. The second zinc center binds in the same manner in this dinuclear dimer structure. In the minor component of (**I**), the acetate group is rotated by 26.7 (16)° (C16—C17 bond *versus* C16*A*—C17*A*) compared to the acetate group in the major component (Fig. 2 ▸), so that both O atoms can coordinate with Zn1 [O2*A*^i^—Zn1—O1*A*^i^ = 54.7 (6)°]. Atom O1*A*^i^ coordinates further with Zn1^i^. The angle made by this distinctive bond, Zn1—O1*A*^i^—Zn1^i^ is 125.1 (8)°. A comparable but different mode of coordination is seen in (**II**): one O atom of an acetate group bridges the two zinc centers with no involvement of the other O atom in the group and the second acetate group shows the same bonding mode by symmetry [Zn1—O—Zn1^i^ = 101.26 (7)°].

## 3. Supra­molecular features

There is an abundance of donor atoms in both structures. However, we found only three hydrogen bonds (Table 3 ▸) in complex (**I**) and none in complex (**II**). These contribute subtly but significantly to packing in both the major and minor disorder components of (**I**). In the minor domain, hydrogen atom H14 from a terminal methyl group (C14) inter­acts with the sulfur atom of an adjacent mol­ecule; the same H atom forms a hydrogen bond with an O atom of an acetate bridging group. In the case of the major domain, H14 in the same manner inter­acts with sulfur atom S1 of an adjacent mol­ecule; additionally, hydrogen atom H17 from the methyl group carbon C17 of the bridging acetate anion reaches to O atom (O1) of an acetate bridging group in a nearby mol­ecule in the major domain configuration. Carbon has a low electronegativity value and is typically not considered a hydrogen-bond donor in the same regard as oxygen, nitro­gen or fluorine. However, these carbon hydrogen-bond donors (C14 and C17) are connected to amide N and acetate –COO^−^ groups, respectively and contribute weak but significant inter­actions. The arrangement and cohesion of mol­ecules in the structure of complex (**I**) does not depend solely on hydrogen bonds. The packing scheme reveals favorable inter­actions between phenyl rings and the aliphatic stem of neighboring mol­ecules contributing favorable van der Waals inter­actions and weak dispersive forces.

**Figure 5:**
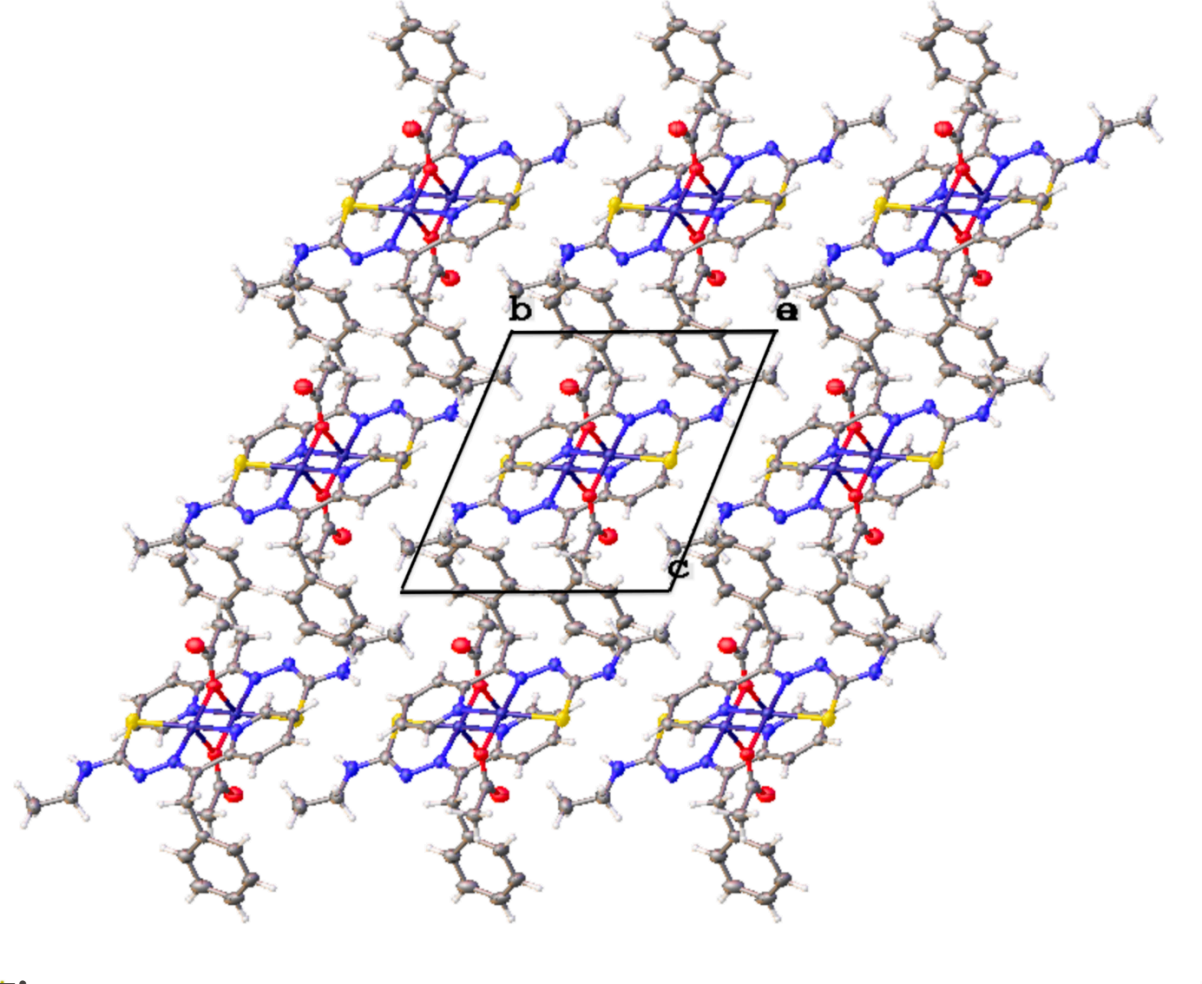
Packing structure of (**II**). A view down [100] is shown along with the unit cell.

It is notable that, even with the abundance of electron donors (hanging double-bonded O atom from the bridging group) and N and S donors from the ligand (Fig. 5 ▸), no hydrogen bonds are found in the extended structure of complex (**II**). Fig. 5 ▸ depicts packing in the crystal and shows a view down [100]. There are no electron acceptors in the vicinity of the O donors. The packing scheme shows additive alignment of hydro­phobic groups (phenyl and aliphatic groups) in addition to potential dispersive forces. The distance between a terminal methyl group and a phenyl ring is 4.54 Å. There is an abundance of –CH_3_ and –CH groups near the exposed double-bonded O atom that can contribute dispersive forces to packing. We detected no aromatic π–π stacking inter­actions. Though there is an abundance of donor groups in the starting ligand, the metal-bound complexes may have lipophilic profile and stability values different from the parent ligand, as we observe in this complex (**II**).

## 4. Database survey

A search of the Cambridge Structural Database (CSD, version 5.44, update September 2023; search date: March 14, 2025; Groom *et al.*, 2016 ▸) for structures similar to **L2** yielded no results. A search on **L1** gave 36 unique hits, 11 of which are unbound ligands and 25 are metal-bound complexes. CSD refcode OBUHAW (Jayakumar *et al.*, 2011 ▸) is recognized as ligand **L1** in its unbound form, also JURBUX (Parry *et al.*, 2025 ▸), that we used for our 2:2 zinc complex (**I**) being reported here. Notable in this set is structure RIYMUH (Valdés-Martínez *et al.*, 1996 ▸), a derivative of OBUHAW that has been evaluated in phase 1 clinical trials for use against cancers (Heffeter *et al.*, 2019 ▸).

The complexes we found in the search are in 1:1, 1:2 or 2:2 metal: ligand ratio and the ligands are tridentate. ARARAM is a centrosymmetric dimer of two monomeric complexes with two chloro groups bridging at the metal centers (Sreekanth & Kurup, 2003 ▸). ARAREQ is similar but it is a monomeric complex, with bromide instead of chloride (Sreekanth & Kurup, 2003 ▸). Coordination around the Cu center is square planar. A 1:2 Cu complex forms in AWEQUQ (Stacy *et al.*, 2016 ▸), where the single positive charge at the copper center is balanced by the perchlorate anion ClO_4_^−^. BIHSIX (Fang *et al.*, 2018 ▸) is a 1:2 complex of zinc and **L1**. In BIHSIX, **L1** is tridentate and coordinates with zinc at the **L1** imine N, pyridine N and sulfur S atoms as in our structure (**I**); the second ligand in BIHSIX binds in the same manner. However, in distinct contrast with BIHSIX, our structure (**I**) is a 2:2 (dinuclear) dimer, though BIHSIX and our (**I**) complex both formed in space group *P*2_1_/*c*.

It is the structure BOFKIS (Jayakumar *et al.*, 2014 ▸), a complex of **L1** with bound copper, to make a dinuclear dimer bridged by two acetate moiety O atoms, that best approximates how zinc is coordinated in our structures, specifically, the major domain of complex (**I**) (Fig. 1 ▸*a*). The other complexes that the search gave are of uncommon metals such as vanadium (DEMKEM; Sreekanth *et al.*, 2006 ▸) and gold (QALDAJ; Sreekanth *et al.*, 2004 ▸).

## 5. Synthesis and crystallization

The ligands **L1** and **L2** were synthesized for us by Enamine LLC (Monmouth Junction, New Jersey, USA) as >95% pure. The zinc-bound complexes of the ligands were obtained by incubating the ligands in a suitable solvent with zinc acetate. We obtained diffraction-quality crystals by vapor diffusion from aceto­nitrile [solvent for structure (**I**)] and from acetone [for structure (**II**)]. In either case we used diethyl ether as precipitant. Crystals were harvested from the vial and trimmed.

## 6. Refinement

Crystal data, data collection and structure refinement details are summarized in Table 4 ▸. We encountered non-merohedral twinning in the diffraction dataset from complex (**I**), and we did not merge the data, hence no *R*_int_ value is reported for this dataset. Hydrogen atoms were placed and allowed to refine using a riding model.

## Supplementary Material

Crystal structure: contains datablock(s) I, II. DOI: 10.1107/S2056989025005407/hb8133sup1.cif

Structure factors: contains datablock(s) I. DOI: 10.1107/S2056989025005407/hb8133Isup4.hkl

Supporting information file. DOI: 10.1107/S2056989025005407/hb8133Isup6.cml

Structure factors: contains datablock(s) II. DOI: 10.1107/S2056989025005407/hb8133IIsup5.hkl

Supporting information file. DOI: 10.1107/S2056989025005407/hb8133IIsup7.cml

CCDC references: 2427149, 2428884

Additional supporting information:  crystallographic information; 3D view; checkCIF report

## Figures and Tables

**Table 1:** Selected bond lengths (Å) for (**I**)

Zn1—S1	2.3705 (17)	Zn1—O2^i^	2.006 (5)
Zn1—N1	2.117 (3)	Zn1—O1*A*	2.114 (14)
Zn1—N4	2.159 (5)	Zn1—O1*A*^i^	2.052 (18)
Zn1—O1	2.018 (5)	Zn1—O2*A*^i^	2.410 (18)

Symmetry code: (i) 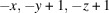.

**Table 2:** Selected bond lengths (Å) for (**II**)

Zn1—S1	2.3360 (6)	Zn1—N1	2.1017 (19)
Zn1—O1^i^	2.0547 (17)	Zn1—N3	2.1128 (19)
Zn1—O1	2.0567 (16)		

Symmetry code: (i) 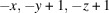.

**Table 3:** Hydrogen-bond geometry (Å, °) for (**I**)

*D*—H⋯*A*	*D*—H	H⋯*A*	*D*⋯*A*	*D*—H⋯*A*
C14—H14*B*⋯S1^ii^	0.96	3.00	3.832 (7)	146
C14—H14*B*⋯O2*A*^iii^	0.96	2.65	3.36 (2)	131
C17—H17*C*⋯O1^iv^	0.96	2.57	3.493 (9)	160

Symmetry codes: (ii) 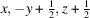; (iii) 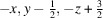; (iv) 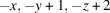.

**Table 4:** Experimental details

	(**I**)	(**II**)
Crystal data		
Chemical formula	[Zn_2_(C_15_H_15_N_4_S)_2_(C_2_H_3_O_2_)_2_]	[Zn_2_(C_16_H_17_N_4_S)_2_(C_2_H_3_O_2_)_2_]
*M*_r_	815.57	843.62
Crystal system, space group	Monoclinic, *P*2_1_/*c*	Triclinic, *P* 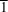
Temperature (K)	296	100
*a*, *b*, *c* (Å)	10.8384 (1), 20.0381 (2), 8.2914 (1)	9.3280 (2), 9.9979 (2), 10.5761 (2)
α, β, γ (°)	90, 91.342 (1), 90	67.101 (2), 83.267 (2), 87.150 (1)
*V* (Å^3^)	1800.24 (3)	902.33 (3)
*Z*	2	1
Radiation type	Cu *K*α	Cu *K*α
μ (mm^−1^)	3.13	3.15
Crystal size (mm)	0.16 × 0.08 × 0.06	0.6 × 0.1 × 0.1

Data collection		
Diffractometer	XtaLAB Synergy, Dualflex, HyPix	XtaLAB Synergy, Dualflex, HyPix
Absorption correction	Gaussian (*CrysAlis PRO*; Rigaku OD, 2022 ▸)	Gaussian (*CrysAlis PRO*; Rigaku OD, 2022 ▸)
*T*_min_, *T*_max_	0.736, 0.849	0.761, 1.000
No. of measured, independent and observed [*I* > 2σ(*I*)] reflections	8417, 8417, 7097	17092, 3599, 3314
*R*_int_	?	0.037
(sin θ/λ)_max_ (Å^−1^)	0.630	0.629

Refinement		
*R*[*F*^2^ > 2σ(*F*^2^)], *wR*(*F*^2^), *S*	0.069, 0.217, 1.05	0.035, 0.099, 1.07
No. of reflections	8417	3599
No. of parameters	244	237
No. of restraints	6	0
H-atom treatment	H-atom parameters constrained	H-atom parameters constrained
Δρ_max_, Δρ_min_ (e Å^−3^)	0.57, −1.01	1.04, −0.61

Computer programs: *CrysAlis PRO* 1.171.42.49 (Rigaku OD, 2022 ▸), *CrysAlis PRO* 1.171.43.91a (Rigaku OD, 2023 ▸), *SHELXT* (Sheldrick, 2015*a* ▸), *SHELXL2019/2* (Sheldrick, 2015*b* ▸), *SHELXL2019/2* (Sheldrick, 2015*b* ▸) and *OLEX2* (Dolomanov *et al.*, 2009 ▸).

## supplementary crystallographic information

### Di-µ-acetato-bis({N,N-dimethyl-2-[phenyl(pyridin-2-yl)methylidene]hydrazine-1-carbothioamidato}zinc(II)) (I) Crystal data

**Table:**

[Zn_2_(C_15_H_15_N_4_S)_2_(C_2_H_3_O_2_)_2_]	*F*(000) = 840
*M**_r_* = 815.57	*D*_x_ = 1.505 Mg m^−^^3^
Monoclinic, *P*2_1_/*c*	Cu *K*α radiation, λ = 1.54184 Å
*a* = 10.8384 (1) Å	Cell parameters from 16893 reflections
*b* = 20.0381 (2) Å	θ = 4.0–75.6°
*c* = 8.2914 (1) Å	µ = 3.13 mm^−^^1^
β = 91.342 (1)°	*T* = 296 K
*V* = 1800.24 (3) Å^3^	Prism, orange
*Z* = 2	0.16 × 0.08 × 0.06 mm

### Di-µ-acetato-bis({N,N-dimethyl-2-[phenyl(pyridin-2-yl)methylidene]hydrazine-1-carbothioamidato}zinc(II)) (I) Data collection

**Table:**

XtaLAB Synergy, Dualflex, HyPix diffractometer	8417 measured reflections
Radiation source: micro-focus sealed X-ray tube, PhotonJet (Cu) X-ray Source	8417 independent reflections
Mirror monochromator	7097 reflections with *I* > 2σ(*I*)
Detector resolution: 10.0000 pixels mm^-1^	θ_max_ = 76.3°, θ_min_ = 4.1°
ω scans	*h* = −13→13
Absorption correction: gaussian (CrysAlisPro; Rigaku OD, 2022)	*k* = −24→25
*T*_min_ = 0.736, *T*_max_ = 0.849	*l* = −10→10

### Di-µ-acetato-bis({N,N-dimethyl-2-[phenyl(pyridin-2-yl)methylidene]hydrazine-1-carbothioamidato}zinc(II)) (I) Refinement

**Table:**

Refinement on *F*^2^	Primary atom site location: dual
Least-squares matrix: full	Hydrogen site location: inferred from neighbouring sites
*R*[*F*^2^ > 2σ(*F*^2^)] = 0.069	H-atom parameters constrained
*wR*(*F*^2^) = 0.217	*w* = 1/[σ^2^(*F*_o_^2^) + (0.1154*P*)^2^ + 1.5821*P*] where *P* = (*F*_o_^2^ + 2*F*_c_^2^)/3
*S* = 1.05	(Δ/σ)_max_ = 0.001
8417 reflections	Δρ_max_ = 0.57 e Å^−^^3^
244 parameters	Δρ_min_ = −1.01 e Å^−^^3^
6 restraints	

### Di-µ-acetato-bis({N,N-dimethyl-2-[phenyl(pyridin-2-yl)methylidene]hydrazine-1-carbothioamidato}zinc(II)) (I) Special details

**Table:**

Geometry. All esds (except the esd in the dihedral angle between two l.s. planes) are estimated using the full covariance matrix. The cell esds are taken into account individually in the estimation of esds in distances, angles and torsion angles; correlations between esds in cell parameters are only used when they are defined by crystal symmetry. An approximate (isotropic) treatment of cell esds is used for estimating esds involving l.s. planes.
Refinement. Refined as a 2-component twin.

### Di-µ-acetato-bis({N,N-dimethyl-2-[phenyl(pyridin-2-yl)methylidene]hydrazine-1-carbothioamidato}zinc(II)) (I) Fractional atomic coordinates and isotropic or equivalent isotropic displacement parameters (Å^2^)

**Table:**

	*x*	*y*	*z*	*U*_iso_*/*U*_eq_	Occ. (<1)
Zn1	−0.12170 (6)	0.44522 (3)	0.57934 (7)	0.0620 (3)	
S1	−0.01854 (11)	0.34367 (8)	0.5248 (2)	0.0798 (4)	
N1	−0.2602 (3)	0.37741 (17)	0.6499 (4)	0.0536 (8)	
N2	−0.2478 (3)	0.31082 (16)	0.6317 (5)	0.0547 (8)	
N3	−0.1212 (4)	0.2251 (2)	0.5758 (6)	0.0748 (12)	
N4	−0.2609 (5)	0.50729 (19)	0.6857 (5)	0.0677 (10)	
C1	−0.3636 (5)	0.4755 (2)	0.7234 (5)	0.0601 (10)	
C2	−0.4601 (6)	0.5100 (3)	0.7907 (8)	0.0817 (16)	
H2	−0.532393	0.487759	0.816378	0.098*	
C3	−0.4488 (9)	0.5777 (3)	0.8195 (9)	0.101 (2)	
H3	−0.513822	0.601271	0.863438	0.122*	
C4	−0.3429 (9)	0.6096 (3)	0.7837 (8)	0.099 (2)	
H4	−0.333412	0.654999	0.803916	0.119*	
C5	−0.2483 (8)	0.5725 (3)	0.7156 (7)	0.0875 (19)	
H5	−0.174815	0.593646	0.690438	0.105*	
C6	−0.4746 (4)	0.3618 (2)	0.7337 (5)	0.0530 (9)	
C7	−0.4657 (5)	0.3067 (2)	0.8357 (6)	0.0615 (10)	
H7	−0.389071	0.293653	0.877574	0.074*	
C8	−0.5704 (6)	0.2714 (3)	0.8748 (8)	0.0812 (17)	
H8	−0.564362	0.235186	0.944869	0.097*	
C9	−0.6835 (6)	0.2896 (4)	0.8104 (11)	0.101 (3)	
H9	−0.753922	0.266196	0.838511	0.121*	
C10	−0.6930 (5)	0.3419 (4)	0.7054 (10)	0.094 (2)	
H10	−0.769334	0.352862	0.658977	0.113*	
C11	−0.5889 (5)	0.3791 (3)	0.6674 (7)	0.0726 (13)	
H11	−0.596052	0.415381	0.597929	0.087*	
C12	−0.3637 (4)	0.4022 (2)	0.6978 (5)	0.0523 (9)	
C13	−0.1374 (4)	0.2914 (2)	0.5811 (5)	0.0606 (10)	
C14	−0.2110 (6)	0.1788 (3)	0.6350 (8)	0.0811 (15)	
H14A	−0.280989	0.202972	0.673169	0.122*	
H14B	−0.174896	0.153228	0.721951	0.122*	
H14C	−0.236959	0.149318	0.549520	0.122*	
C15	−0.0079 (7)	0.1946 (4)	0.5192 (11)	0.107 (2)	
H15A	0.046817	0.186037	0.609514	0.161*	
H15B	0.031277	0.224407	0.445524	0.161*	
H15C	−0.027236	0.153412	0.465239	0.161*	
O1	−0.0062 (5)	0.4927 (4)	0.7356 (7)	0.0946 (18)	0.756 (6)
O2	0.1716 (5)	0.5131 (3)	0.6323 (6)	0.0787 (15)	0.756 (6)
C16	0.1073 (7)	0.4970 (7)	0.7512 (8)	0.0500 (16)	0.756 (6)
C17	0.1712 (9)	0.4881 (5)	0.9105 (9)	0.076 (2)	0.756 (6)
H17A	0.246371	0.513441	0.913051	0.114*	0.756 (6)
H17B	0.189858	0.441711	0.926914	0.114*	0.756 (6)
H17C	0.118602	0.503386	0.994452	0.114*	0.756 (6)
O1A	0.0452 (14)	0.4995 (11)	0.601 (2)	0.0946 (18)	0.244 (6)
O2A	0.2194 (16)	0.5302 (11)	0.678 (2)	0.0787 (15)	0.244 (6)
C16A	0.120 (3)	0.502 (2)	0.715 (4)	0.0500 (16)	0.244 (6)
C17A	0.122 (3)	0.4713 (16)	0.875 (3)	0.076 (2)	0.244 (6)
H17D	0.202769	0.453691	0.899451	0.114*	0.244 (6)
H17E	0.062579	0.435778	0.877541	0.114*	0.244 (6)
H17F	0.101735	0.504297	0.954302	0.114*	0.244 (6)

### Di-µ-acetato-bis({N,N-dimethyl-2-[phenyl(pyridin-2-yl)methylidene]hydrazine-1-carbothioamidato}zinc(II)) (I) Atomic displacement parameters (Å^2^)

**Table:**

	*U*^11^	*U*^22^	*U*^33^	*U*^12^	*U*^13^	*U*^23^
Zn1	0.0618 (4)	0.0728 (4)	0.0514 (4)	−0.0222 (3)	−0.0024 (3)	0.0105 (2)
S1	0.0484 (6)	0.1006 (10)	0.0910 (10)	−0.0045 (5)	0.0119 (6)	0.0123 (7)
N1	0.0529 (18)	0.0538 (17)	0.0539 (19)	−0.0059 (13)	−0.0001 (15)	0.0022 (14)
N2	0.0465 (17)	0.0509 (17)	0.067 (2)	−0.0003 (13)	0.0030 (15)	0.0026 (14)
N3	0.064 (2)	0.074 (3)	0.087 (3)	0.0191 (18)	0.005 (2)	0.002 (2)
N4	0.097 (3)	0.0536 (19)	0.052 (2)	−0.0192 (18)	−0.0040 (19)	0.0056 (15)
C1	0.080 (3)	0.054 (2)	0.046 (2)	−0.0020 (19)	0.000 (2)	0.0037 (16)
C2	0.105 (4)	0.058 (3)	0.084 (4)	0.005 (3)	0.019 (3)	−0.007 (2)
C3	0.151 (7)	0.059 (3)	0.095 (4)	0.019 (4)	0.018 (4)	−0.004 (3)
C4	0.175 (8)	0.046 (2)	0.077 (4)	−0.006 (3)	0.005 (4)	−0.002 (2)
C5	0.142 (6)	0.057 (3)	0.062 (3)	−0.028 (3)	−0.008 (3)	0.011 (2)
C6	0.049 (2)	0.057 (2)	0.053 (2)	−0.0027 (15)	0.0048 (17)	−0.0061 (16)
C7	0.063 (3)	0.062 (2)	0.059 (3)	−0.0090 (18)	0.006 (2)	0.0016 (18)
C8	0.091 (4)	0.066 (3)	0.088 (4)	−0.021 (3)	0.032 (3)	−0.009 (2)
C9	0.067 (3)	0.096 (4)	0.141 (6)	−0.030 (3)	0.045 (4)	−0.052 (4)
C10	0.047 (2)	0.105 (5)	0.131 (6)	0.000 (3)	0.003 (3)	−0.052 (4)
C11	0.059 (3)	0.078 (3)	0.080 (3)	0.012 (2)	−0.006 (2)	−0.014 (2)
C12	0.052 (2)	0.054 (2)	0.050 (2)	−0.0024 (16)	0.0000 (17)	0.0023 (16)
C13	0.054 (2)	0.070 (3)	0.058 (3)	0.0034 (18)	−0.0021 (19)	0.0053 (19)
C14	0.080 (3)	0.059 (3)	0.103 (4)	0.009 (2)	−0.015 (3)	0.008 (3)
C15	0.087 (4)	0.104 (5)	0.131 (6)	0.040 (4)	0.020 (4)	−0.007 (4)
O1	0.059 (3)	0.153 (5)	0.072 (3)	−0.036 (3)	0.008 (2)	−0.036 (3)
O2	0.051 (3)	0.123 (4)	0.061 (3)	−0.003 (3)	−0.005 (2)	0.024 (3)
C16	0.055 (3)	0.052 (3)	0.043 (4)	0.004 (2)	0.005 (3)	−0.003 (4)
C17	0.081 (6)	0.096 (6)	0.050 (4)	0.011 (4)	0.002 (3)	0.005 (3)
O1A	0.059 (3)	0.153 (5)	0.072 (3)	−0.036 (3)	0.008 (2)	−0.036 (3)
O2A	0.051 (3)	0.123 (4)	0.061 (3)	−0.003 (3)	−0.005 (2)	0.024 (3)
C16A	0.055 (3)	0.052 (3)	0.043 (4)	0.004 (2)	0.005 (3)	−0.003 (4)
C17A	0.081 (6)	0.096 (6)	0.050 (4)	0.011 (4)	0.002 (3)	0.005 (3)

### Di-µ-acetato-bis({N,N-dimethyl-2-[phenyl(pyridin-2-yl)methylidene]hydrazine-1-carbothioamidato}zinc(II)) (I) Geometric parameters (Å, º)

**Table:**

Zn1—S1	2.3705 (17)	C6—C12	1.485 (6)
Zn1—N1	2.117 (3)	C7—H7	0.9300
Zn1—N4	2.159 (5)	C7—C8	1.383 (7)
Zn1—O1	2.018 (5)	C8—H8	0.9300
Zn1—O2^i^	2.006 (5)	C8—C9	1.375 (11)
Zn1—O1A	2.114 (14)	C9—H9	0.9300
Zn1—O1A^i^	2.052 (18)	C9—C10	1.365 (12)
Zn1—O2A^i^	2.410 (18)	C10—H10	0.9300
S1—C13	1.733 (5)	C10—C11	1.394 (9)
N1—N2	1.350 (5)	C11—H11	0.9300
N1—C12	1.298 (6)	C14—H14A	0.9600
N2—C13	1.334 (6)	C14—H14B	0.9600
N3—C13	1.342 (7)	C14—H14C	0.9600
N3—C14	1.438 (8)	C15—H15A	0.9600
N3—C15	1.459 (7)	C15—H15B	0.9600
N4—C1	1.327 (7)	C15—H15C	0.9600
N4—C5	1.336 (7)	O1—C16	1.237 (9)
C1—C2	1.382 (8)	O2—C16	1.262 (8)
C1—C12	1.485 (6)	C16—C17	1.488 (10)
C2—H2	0.9300	C17—H17A	0.9600
C2—C3	1.383 (8)	C17—H17B	0.9600
C3—H3	0.9300	C17—H17C	0.9600
C3—C4	1.352 (11)	O1A—C16A	1.24 (2)
C4—H4	0.9300	O2A—C16A	1.26 (2)
C4—C5	1.397 (11)	C16A—C17A	1.46 (2)
C5—H5	0.9300	C17A—H17D	0.9600
C6—C7	1.392 (6)	C17A—H17E	0.9600
C6—C11	1.387 (7)	C17A—H17F	0.9600
			
S1—Zn1—O2A^i^	101.9 (5)	C6—C7—H7	119.9
N1—Zn1—S1	80.93 (10)	C8—C7—C6	120.2 (5)
N1—Zn1—N4	75.53 (14)	C8—C7—H7	119.9
N1—Zn1—O2A^i^	94.4 (4)	C7—C8—H8	119.9
N4—Zn1—S1	155.44 (10)	C9—C8—C7	120.1 (6)
N4—Zn1—O2A^i^	87.0 (5)	C9—C8—H8	119.9
O1—Zn1—S1	103.8 (2)	C8—C9—H9	119.8
O1—Zn1—N1	123.9 (2)	C10—C9—C8	120.4 (5)
O1—Zn1—N4	84.0 (2)	C10—C9—H9	119.8
O1—Zn1—O1A^i^	87.6 (4)	C9—C10—H10	119.8
O1—Zn1—O2A^i^	136.5 (6)	C9—C10—C11	120.3 (6)
O2^i^—Zn1—S1	108.03 (19)	C11—C10—H10	119.8
O2^i^—Zn1—N1	109.3 (2)	C6—C11—C10	119.8 (6)
O2^i^—Zn1—N4	86.6 (2)	C6—C11—H11	120.1
O2^i^—Zn1—O1	121.0 (3)	C10—C11—H11	120.1
O2^i^—Zn1—O1A^i^	40.8 (5)	N1—C12—C1	115.1 (4)
O2^i^—Zn1—O2A^i^	15.7 (5)	N1—C12—C6	124.2 (4)
O1A—Zn1—S1	92.9 (6)	C1—C12—C6	120.6 (4)
O1A^i^—Zn1—S1	97.1 (6)	N2—C13—S1	125.9 (4)
O1A^i^—Zn1—N1	148.1 (4)	N2—C13—N3	114.7 (4)
O1A—Zn1—N1	156.5 (6)	N3—C13—S1	119.4 (3)
O1A—Zn1—N4	105.9 (5)	N3—C14—H14A	109.5
O1A^i^—Zn1—N4	106.5 (6)	N3—C14—H14B	109.5
O1A^i^—Zn1—O1A	54.9 (8)	N3—C14—H14C	109.5
O1A—Zn1—O2A^i^	109.1 (7)	H14A—C14—H14B	109.5
O1A^i^—Zn1—O2A^i^	54.7 (6)	H14A—C14—H14C	109.5
C13—S1—Zn1	96.33 (16)	H14B—C14—H14C	109.5
N2—N1—Zn1	122.0 (3)	N3—C15—H15A	109.5
C12—N1—Zn1	117.6 (3)	N3—C15—H15B	109.5
C12—N1—N2	120.1 (3)	N3—C15—H15C	109.5
C13—N2—N1	114.6 (3)	H15A—C15—H15B	109.5
C13—N3—C14	122.5 (4)	H15A—C15—H15C	109.5
C13—N3—C15	122.4 (5)	H15B—C15—H15C	109.5
C14—N3—C15	114.9 (5)	C16—O1—Zn1	134.4 (6)
C1—N4—Zn1	114.7 (3)	C16—O2—Zn1^i^	130.3 (6)
C1—N4—C5	120.6 (5)	O1—C16—O2	120.1 (7)
C5—N4—Zn1	124.7 (4)	O1—C16—C17	121.7 (6)
N4—C1—C2	120.2 (4)	O2—C16—C17	118.0 (7)
N4—C1—C12	116.0 (4)	C16—C17—H17A	109.5
C2—C1—C12	123.7 (4)	C16—C17—H17B	109.5
C1—C2—H2	120.2	C16—C17—H17C	109.5
C1—C2—C3	119.7 (6)	H17A—C17—H17B	109.5
C3—C2—H2	120.2	H17A—C17—H17C	109.5
C2—C3—H3	120.0	H17B—C17—H17C	109.5
C4—C3—C2	119.9 (6)	Zn1^i^—O1A—Zn1	125.1 (8)
C4—C3—H3	120.0	C16A—O1A—Zn1	129.3 (18)
C3—C4—H4	120.9	O1A—C16A—O2A	112 (2)
C3—C4—C5	118.1 (5)	O1A—C16A—C17A	132 (3)
C5—C4—H4	120.9	O2A—C16A—C17A	115 (2)
N4—C5—C4	121.5 (6)	C16A—C17A—H17D	109.5
N4—C5—H5	119.2	C16A—C17A—H17E	109.5
C4—C5—H5	119.2	C16A—C17A—H17F	109.5
C7—C6—C12	120.6 (4)	H17D—C17A—H17E	109.5
C11—C6—C7	119.2 (4)	H17D—C17A—H17F	109.5
C11—C6—C12	120.3 (4)	H17E—C17A—H17F	109.5
			
Zn1—S1—C13—N2	1.8 (4)	C1—C2—C3—C4	−0.8 (11)
Zn1—S1—C13—N3	−178.0 (4)	C2—C1—C12—N1	−172.7 (5)
Zn1—N1—N2—C13	6.3 (5)	C2—C1—C12—C6	4.5 (7)
Zn1—N1—C12—C1	−10.1 (5)	C2—C3—C4—C5	1.0 (11)
Zn1—N1—C12—C6	172.9 (3)	C3—C4—C5—N4	0.1 (10)
Zn1—N4—C1—C2	−179.3 (4)	C5—N4—C1—C2	1.6 (8)
Zn1—N4—C1—C12	4.1 (5)	C5—N4—C1—C12	−175.0 (5)
Zn1—N4—C5—C4	179.5 (5)	C6—C7—C8—C9	1.4 (8)
Zn1—O1—C16—O2	51.2 (18)	C7—C6—C11—C10	0.9 (7)
Zn1—O1—C16—C17	−133.0 (9)	C7—C6—C12—N1	50.6 (6)
Zn1^i^—O2—C16—O1	15.9 (18)	C7—C6—C12—C1	−126.3 (5)
Zn1^i^—O2—C16—C17	−160.0 (7)	C7—C8—C9—C10	1.1 (9)
Zn1—O1A—C16A—O2A	169 (2)	C8—C9—C10—C11	−2.6 (9)
Zn1^i^—O1A—C16A—O2A	−7 (5)	C9—C10—C11—C6	1.6 (8)
Zn1—O1A—C16A—C17A	−2 (8)	C11—C6—C7—C8	−2.4 (7)
Zn1^i^—O1A—C16A—C17A	−178 (5)	C11—C6—C12—N1	−130.3 (5)
Zn1^i^—O2A—C16A—O1A	5 (4)	C11—C6—C12—C1	52.8 (6)
Zn1^i^—O2A—C16A—C17A	178 (4)	C12—N1—N2—C13	179.7 (4)
N1—N2—C13—S1	−5.2 (6)	C12—C1—C2—C3	175.9 (6)
N1—N2—C13—N3	174.7 (4)	C12—C6—C7—C8	176.7 (4)
N2—N1—C12—C1	176.3 (4)	C12—C6—C11—C10	−178.2 (4)
N2—N1—C12—C6	−0.8 (6)	C14—N3—C13—S1	173.9 (4)
N4—C1—C2—C3	−0.5 (9)	C14—N3—C13—N2	−5.9 (8)
N4—C1—C12—N1	3.8 (6)	C15—N3—C13—S1	−2.0 (8)
N4—C1—C12—C6	−179.1 (4)	C15—N3—C13—N2	178.2 (6)
C1—N4—C5—C4	−1.4 (8)		

Symmetry code: (i) −*x*, −*y*+1, −*z*+1.

### Di-µ-acetato-bis({N,N-dimethyl-2-[phenyl(pyridin-2-yl)methylidene]hydrazine-1-carbothioamidato}zinc(II)) (I) Hydrogen-bond geometry (Å, º)

**Table:**

*D*—H···*A*	*D*—H	H···*A*	*D*···*A*	*D*—H···*A*
C14—H14*B*···S1^ii^	0.96	3.00	3.832 (7)	146
C14—H14*B*···O2*A*^iii^	0.96	2.65	3.36 (2)	131
C17—H17*C*···O1^iv^	0.96	2.57	3.493 (9)	160

Symmetry codes: (ii) *x*, −*y*+1/2, *z*+1/2; (iii) −*x*, *y*−1/2, −*z*+3/2; (iv) −*x*, −*y*+1, −*z*+2.

### Di-µ-acetato-bis({N-ethyl-2-[phenyl(pyridin-2-yl)methylidene]hydrazine-1-carbothioamidato}zinc(II)) (II) Crystal data

**Table:**

[Zn_2_(C_16_H_17_N_4_S)_2_(C_2_H_3_O_2_)_2_]	*Z* = 1
*M**_r_* = 843.62	*F*(000) = 436
Triclinic, *P*1	*D*_x_ = 1.553 Mg m^−^^3^
*a* = 9.3280 (2) Å	Cu *K*α radiation, λ = 1.54184 Å
*b* = 9.9979 (2) Å	Cell parameters from 10059 reflections
*c* = 10.5761 (2) Å	θ = 4.6–75.5°
α = 67.101 (2)°	µ = 3.15 mm^−^^1^
β = 83.267 (2)°	*T* = 100 K
γ = 87.150 (1)°	Needle, metallic orangish yellow
*V* = 902.33 (3) Å^3^	0.6 × 0.1 × 0.1 mm

### Di-µ-acetato-bis({N-ethyl-2-[phenyl(pyridin-2-yl)methylidene]hydrazine-1-carbothioamidato}zinc(II)) (II) Data collection

**Table:**

XtaLAB Synergy, Dualflex, HyPix diffractometer	3599 independent reflections
Radiation source: micro-focus sealed X-ray tube, PhotonJet (Cu) X-ray Source	3314 reflections with *I* > 2σ(*I*)
Mirror monochromator	*R*_int_ = 0.037
Detector resolution: 10.0000 pixels mm^-1^	θ_max_ = 75.9°, θ_min_ = 4.6°
ω scans	*h* = −11→11
Absorption correction: gaussian (CrysAlisPro; Rigaku OD, 2022)	*k* = −12→12
*T*_min_ = 0.761, *T*_max_ = 1.000	*l* = −13→8
17092 measured reflections	

### Di-µ-acetato-bis({N-ethyl-2-[phenyl(pyridin-2-yl)methylidene]hydrazine-1-carbothioamidato}zinc(II)) (II) Refinement

**Table:**

Refinement on *F*^2^	Primary atom site location: dual
Least-squares matrix: full	Hydrogen site location: inferred from neighbouring sites
*R*[*F*^2^ > 2σ(*F*^2^)] = 0.035	H-atom parameters constrained
*wR*(*F*^2^) = 0.099	*w* = 1/[σ^2^(*F*_o_^2^) + (0.0496*P*)^2^ + 0.808*P*] where *P* = (*F*_o_^2^ + 2*F*_c_^2^)/3
*S* = 1.07	(Δ/σ)_max_ = 0.001
3599 reflections	Δρ_max_ = 1.04 e Å^−^^3^
237 parameters	Δρ_min_ = −0.61 e Å^−^^3^
0 restraints	

### Di-µ-acetato-bis({N-ethyl-2-[phenyl(pyridin-2-yl)methylidene]hydrazine-1-carbothioamidato}zinc(II)) (II) Special details

**Table:**

Geometry. All esds (except the esd in the dihedral angle between two l.s. planes) are estimated using the full covariance matrix. The cell esds are taken into account individually in the estimation of esds in distances, angles and torsion angles; correlations between esds in cell parameters are only used when they are defined by crystal symmetry. An approximate (isotropic) treatment of cell esds is used for estimating esds involving l.s. planes.
Refinement. n/a

### Di-µ-acetato-bis({N-ethyl-2-[phenyl(pyridin-2-yl)methylidene]hydrazine-1-carbothioamidato}zinc(II)) (II) Fractional atomic coordinates and isotropic or equivalent isotropic displacement parameters (Å^2^)

**Table:**

	*x*	*y*	*z*	*U*_iso_*/*U*_eq_	
Zn1	0.13763 (3)	0.56946 (3)	0.52595 (3)	0.02666 (12)	
S1	0.07920 (7)	0.80673 (6)	0.50446 (7)	0.03498 (16)	
O1	0.03886 (17)	0.55584 (18)	0.36744 (17)	0.0314 (4)	
O2	0.20742 (19)	0.6904 (2)	0.2109 (2)	0.0420 (4)	
N1	0.2769 (2)	0.5869 (2)	0.66120 (19)	0.0257 (4)	
N2	0.2572 (2)	0.6924 (2)	0.7140 (2)	0.0291 (4)	
N3	0.2913 (2)	0.4029 (2)	0.54080 (19)	0.0267 (4)	
C1	0.3747 (2)	0.4885 (2)	0.7032 (2)	0.0261 (5)	
C2	0.1670 (2)	0.7967 (2)	0.6451 (2)	0.0294 (5)	
N5	0.1441 (2)	0.9069 (2)	0.6877 (2)	0.0350 (5)	
H5	0.081887	0.974927	0.647316	0.042*	
C4	0.2163 (3)	0.9198 (3)	0.7967 (3)	0.0380 (6)	
H4A	0.320567	0.937002	0.766181	0.046*	
H4B	0.205478	0.828339	0.879667	0.046*	
C5	0.1511 (4)	1.0449 (3)	0.8317 (3)	0.0474 (7)	
H5A	0.166587	1.135871	0.750695	0.071*	
H5B	0.197324	1.050581	0.908014	0.071*	
H5C	0.047298	1.028886	0.859057	0.071*	
C6	0.3890 (2)	0.3859 (2)	0.6315 (2)	0.0243 (4)	
C7	0.4979 (2)	0.2824 (2)	0.6496 (2)	0.0274 (5)	
H7	0.566977	0.272140	0.712077	0.033*	
C8	0.5045 (2)	0.1939 (2)	0.5752 (2)	0.0292 (5)	
H8	0.578194	0.122378	0.586622	0.035*	
C9	0.4035 (3)	0.2103 (3)	0.4844 (2)	0.0305 (5)	
H9	0.405956	0.150358	0.433217	0.037*	
C10	0.2986 (3)	0.3169 (3)	0.4703 (2)	0.0298 (5)	
H10	0.229027	0.329140	0.407845	0.036*	
C11	0.4011 (3)	0.3593 (3)	0.9519 (2)	0.0299 (5)	
C12	0.4561 (3)	0.2191 (3)	1.0019 (3)	0.0428 (6)	
H12	0.540830	0.195757	0.955343	0.051*	
C13	0.3887 (4)	0.1127 (3)	1.1191 (3)	0.0528 (8)	
H13	0.426549	0.016658	1.151237	0.063*	
C14	0.2658 (3)	0.1459 (4)	1.1899 (3)	0.0489 (7)	
H14	0.217569	0.072526	1.268490	0.059*	
C15	0.2151 (3)	0.2871 (4)	1.1442 (3)	0.0478 (7)	
H15	0.133473	0.311632	1.193703	0.057*	
C16	0.2825 (3)	0.3938 (3)	1.0261 (3)	0.0377 (6)	
H16	0.247096	0.490733	0.996116	0.045*	
C17	0.4658 (2)	0.4735 (3)	0.8165 (2)	0.0290 (5)	
H17A	0.470220	0.567857	0.825991	0.035*	
H17B	0.565358	0.444870	0.793100	0.035*	
C18	0.0920 (2)	0.6274 (3)	0.2411 (2)	0.0298 (5)	
C19	0.0001 (3)	0.6270 (3)	0.1320 (3)	0.0386 (6)	
H19A	−0.021844	0.526672	0.147366	0.058*	
H19B	0.052749	0.674272	0.040290	0.058*	
H19C	−0.090138	0.679880	0.137940	0.058*	

### Di-µ-acetato-bis({N-ethyl-2-[phenyl(pyridin-2-yl)methylidene]hydrazine-1-carbothioamidato}zinc(II)) (II) Atomic displacement parameters (Å^2^)

**Table:**

	*U*^11^	*U*^22^	*U*^33^	*U*^12^	*U*^13^	*U*^23^
Zn1	0.02338 (17)	0.02572 (18)	0.02823 (18)	0.00230 (12)	−0.00559 (12)	−0.00704 (13)
S1	0.0350 (3)	0.0272 (3)	0.0430 (3)	0.0064 (2)	−0.0152 (3)	−0.0116 (3)
O1	0.0300 (8)	0.0344 (9)	0.0279 (8)	0.0010 (7)	−0.0068 (7)	−0.0090 (7)
O2	0.0272 (9)	0.0545 (12)	0.0426 (10)	−0.0061 (8)	−0.0036 (7)	−0.0160 (9)
N1	0.0243 (9)	0.0238 (9)	0.0266 (9)	−0.0006 (7)	−0.0023 (7)	−0.0072 (8)
N2	0.0277 (9)	0.0264 (10)	0.0331 (10)	−0.0004 (7)	−0.0029 (8)	−0.0114 (8)
N3	0.0224 (9)	0.0263 (9)	0.0283 (9)	0.0003 (7)	−0.0036 (7)	−0.0069 (8)
C1	0.0211 (10)	0.0276 (11)	0.0254 (11)	−0.0028 (8)	0.0012 (8)	−0.0063 (9)
C2	0.0265 (11)	0.0249 (11)	0.0342 (12)	−0.0021 (9)	0.0014 (9)	−0.0096 (9)
N5	0.0344 (11)	0.0277 (10)	0.0427 (12)	0.0028 (8)	−0.0059 (9)	−0.0132 (9)
C4	0.0404 (14)	0.0359 (13)	0.0375 (13)	−0.0054 (11)	−0.0013 (11)	−0.0142 (11)
C5	0.0574 (18)	0.0422 (15)	0.0453 (16)	−0.0035 (13)	0.0029 (13)	−0.0216 (13)
C6	0.0202 (10)	0.0241 (10)	0.0238 (10)	−0.0016 (8)	−0.0016 (8)	−0.0041 (8)
C7	0.0234 (10)	0.0272 (11)	0.0263 (11)	−0.0016 (8)	−0.0017 (8)	−0.0046 (9)
C8	0.0254 (11)	0.0259 (11)	0.0307 (11)	0.0020 (9)	0.0003 (9)	−0.0062 (9)
C9	0.0311 (12)	0.0308 (12)	0.0303 (12)	−0.0016 (9)	−0.0016 (9)	−0.0129 (10)
C10	0.0285 (11)	0.0310 (12)	0.0298 (11)	−0.0015 (9)	−0.0052 (9)	−0.0107 (10)
C11	0.0286 (11)	0.0360 (12)	0.0263 (11)	−0.0004 (9)	−0.0085 (9)	−0.0118 (10)
C12	0.0521 (16)	0.0403 (15)	0.0295 (13)	0.0093 (12)	−0.0064 (11)	−0.0069 (11)
C13	0.076 (2)	0.0392 (16)	0.0337 (14)	0.0041 (15)	−0.0108 (14)	−0.0030 (12)
C14	0.0525 (17)	0.0581 (19)	0.0267 (13)	−0.0123 (14)	−0.0095 (12)	−0.0032 (12)
C15	0.0347 (14)	0.072 (2)	0.0317 (13)	−0.0006 (13)	−0.0037 (11)	−0.0143 (14)
C16	0.0329 (13)	0.0474 (15)	0.0316 (12)	0.0021 (11)	−0.0061 (10)	−0.0135 (11)
C17	0.0254 (11)	0.0322 (12)	0.0288 (11)	0.0007 (9)	−0.0051 (9)	−0.0105 (10)
C18	0.0246 (11)	0.0317 (12)	0.0311 (12)	0.0041 (9)	−0.0028 (9)	−0.0105 (10)
C19	0.0358 (13)	0.0487 (15)	0.0290 (12)	−0.0040 (11)	−0.0029 (10)	−0.0122 (11)

### Di-µ-acetato-bis({N-ethyl-2-[phenyl(pyridin-2-yl)methylidene]hydrazine-1-carbothioamidato}zinc(II)) (II) Geometric parameters (Å, º)

**Table:**

Zn1—S1	2.3360 (6)	C7—H7	0.9500
Zn1—O1^i^	2.0547 (17)	C7—C8	1.390 (3)
Zn1—O1	2.0567 (16)	C8—H8	0.9500
Zn1—N1	2.1017 (19)	C8—C9	1.381 (3)
Zn1—N3	2.1128 (19)	C9—H9	0.9500
S1—C2	1.748 (3)	C9—C10	1.386 (3)
O1—C18	1.296 (3)	C10—H10	0.9500
O2—C18	1.220 (3)	C11—C12	1.386 (4)
N1—N2	1.369 (3)	C11—C16	1.389 (4)
N1—C1	1.290 (3)	C11—C17	1.521 (3)
N2—C2	1.338 (3)	C12—H12	0.9500
N3—C6	1.358 (3)	C12—C13	1.385 (4)
N3—C10	1.335 (3)	C13—H13	0.9500
C1—C6	1.488 (3)	C13—C14	1.393 (5)
C1—C17	1.506 (3)	C14—H14	0.9500
C2—N5	1.342 (3)	C14—C15	1.380 (5)
N5—H5	0.8800	C15—H15	0.9500
N5—C4	1.451 (3)	C15—C16	1.392 (4)
C4—H4A	0.9900	C16—H16	0.9500
C4—H4B	0.9900	C17—H17A	0.9900
C4—C5	1.518 (4)	C17—H17B	0.9900
C5—H5A	0.9800	C18—C19	1.517 (3)
C5—H5B	0.9800	C19—H19A	0.9800
C5—H5C	0.9800	C19—H19B	0.9800
C6—C7	1.388 (3)	C19—H19C	0.9800
			
O1—Zn1—S1	101.39 (5)	C6—C7—H7	120.5
O1^i^—Zn1—S1	106.55 (5)	C6—C7—C8	119.1 (2)
O1^i^—Zn1—O1	78.74 (7)	C8—C7—H7	120.5
O1^i^—Zn1—N1	111.25 (7)	C7—C8—H8	120.1
O1—Zn1—N1	168.46 (7)	C9—C8—C7	119.8 (2)
O1^i^—Zn1—N3	99.24 (7)	C9—C8—H8	120.1
O1—Zn1—N3	96.75 (7)	C8—C9—H9	121.0
N1—Zn1—S1	81.70 (5)	C8—C9—C10	118.1 (2)
N1—Zn1—N3	76.34 (7)	C10—C9—H9	121.0
N3—Zn1—S1	150.82 (6)	N3—C10—C9	122.7 (2)
C2—S1—Zn1	94.80 (8)	N3—C10—H10	118.6
Zn1^i^—O1—Zn1	101.26 (7)	C9—C10—H10	118.6
C18—O1—Zn1	119.22 (15)	C12—C11—C16	118.8 (2)
C18—O1—Zn1^i^	139.34 (15)	C12—C11—C17	121.3 (2)
N2—N1—Zn1	121.20 (14)	C16—C11—C17	119.9 (2)
C1—N1—Zn1	118.57 (16)	C11—C12—H12	119.6
C1—N1—N2	119.98 (19)	C13—C12—C11	120.8 (3)
C2—N2—N1	111.85 (19)	C13—C12—H12	119.6
C6—N3—Zn1	114.71 (15)	C12—C13—H13	119.9
C10—N3—Zn1	125.89 (16)	C12—C13—C14	120.3 (3)
C10—N3—C6	119.41 (19)	C14—C13—H13	119.9
N1—C1—C6	114.0 (2)	C13—C14—H14	120.5
N1—C1—C17	124.3 (2)	C15—C14—C13	119.0 (3)
C6—C1—C17	121.69 (19)	C15—C14—H14	120.5
N2—C2—S1	127.26 (18)	C14—C15—H15	119.7
N2—C2—N5	116.0 (2)	C14—C15—C16	120.6 (3)
N5—C2—S1	116.74 (18)	C16—C15—H15	119.7
C2—N5—H5	118.2	C11—C16—C15	120.4 (3)
C2—N5—C4	123.5 (2)	C11—C16—H16	119.8
C4—N5—H5	118.2	C15—C16—H16	119.8
N5—C4—H4A	109.7	C1—C17—C11	109.73 (18)
N5—C4—H4B	109.7	C1—C17—H17A	109.7
N5—C4—C5	109.7 (2)	C1—C17—H17B	109.7
H4A—C4—H4B	108.2	C11—C17—H17A	109.7
C5—C4—H4A	109.7	C11—C17—H17B	109.7
C5—C4—H4B	109.7	H17A—C17—H17B	108.2
C4—C5—H5A	109.5	O1—C18—C19	115.0 (2)
C4—C5—H5B	109.5	O2—C18—O1	123.0 (2)
C4—C5—H5C	109.5	O2—C18—C19	122.0 (2)
H5A—C5—H5B	109.5	C18—C19—H19A	109.5
H5A—C5—H5C	109.5	C18—C19—H19B	109.5
H5B—C5—H5C	109.5	C18—C19—H19C	109.5
N3—C6—C1	115.76 (19)	H19A—C19—H19B	109.5
N3—C6—C7	120.8 (2)	H19A—C19—H19C	109.5
C7—C6—C1	123.3 (2)	H19B—C19—H19C	109.5
			
Zn1—S1—C2—N2	11.2 (2)	C1—N1—N2—C2	170.9 (2)
Zn1—S1—C2—N5	−170.12 (17)	C1—C6—C7—C8	−178.3 (2)
Zn1—O1—C18—O2	8.3 (3)	C2—N5—C4—C5	−172.4 (2)
Zn1^i^—O1—C18—O2	−177.72 (18)	C6—N3—C10—C9	−0.6 (3)
Zn1^i^—O1—C18—C19	2.2 (4)	C6—C1—C17—C11	−81.4 (2)
Zn1—O1—C18—C19	−171.85 (16)	C6—C7—C8—C9	0.2 (3)
Zn1—N1—N2—C2	−15.0 (2)	C7—C8—C9—C10	0.5 (3)
Zn1—N1—C1—C6	8.8 (2)	C8—C9—C10—N3	−0.3 (3)
Zn1—N1—C1—C17	−169.86 (16)	C10—N3—C6—C1	178.71 (19)
Zn1—N3—C6—C1	−1.3 (2)	C10—N3—C6—C7	1.3 (3)
Zn1—N3—C6—C7	−178.70 (16)	C11—C12—C13—C14	−1.1 (5)
Zn1—N3—C10—C9	179.39 (17)	C12—C11—C16—C15	−3.7 (4)
S1—C2—N5—C4	−175.73 (18)	C12—C11—C17—C1	100.0 (3)
N1—N2—C2—S1	0.3 (3)	C12—C13—C14—C15	−2.0 (5)
N1—N2—C2—N5	−178.42 (19)	C13—C14—C15—C16	2.3 (4)
N1—C1—C6—N3	−4.8 (3)	C14—C15—C16—C11	0.6 (4)
N1—C1—C6—C7	172.5 (2)	C16—C11—C12—C13	4.0 (4)
N1—C1—C17—C11	97.2 (3)	C16—C11—C17—C1	−78.4 (3)
N2—N1—C1—C6	−176.93 (18)	C17—C1—C6—N3	173.91 (19)
N2—N1—C1—C17	4.4 (3)	C17—C1—C6—C7	−8.8 (3)
N2—C2—N5—C4	3.1 (3)	C17—C11—C12—C13	−174.4 (3)
N3—C6—C7—C8	−1.1 (3)	C17—C11—C16—C15	174.7 (2)

Symmetry code: (i) −*x*, −*y*+1, −*z*+1.
